# *GRK5* Intronic (CA)_n_ Polymorphisms Associated with Type 2 Diabetes in Chinese Hainan Island

**DOI:** 10.1371/journal.pone.0090597

**Published:** 2014-03-03

**Authors:** Zhenfang Xia, Tubao Yang, Zhuansuo Wang, Jianping Dong, Chunyan Liang

**Affiliations:** 1 Division of Health Statistics, School of Public Health, Central South University, City of Changsha, Province Hunan, China; 2 Department of Endocrinology, the Affiliated Hospital of Hainan Medical College, City of Haikou, Province Hainan, China; 3 Transgenic Laboratory, Hainan Medical College, City of Haikou, Province Hainan, China; 4 Outpatients Department, Community Health Service Center of Xiuying, City of Haikou, Province Hainan, China; University of Warwick – Medical School, United Kingdom

## Abstract

A genome-wide association study had showed G-protein–coupled receptor kinase 5 (*GRK5*) rs10886471 was related to the risk of type 2 diabetes mellitus (T2DM) through upregulated *GRK5* mRNA expression. Rs10886471 is located in the intron region of *GRK5*. However, the mechanism by which intronic SNP affects gene expression remains unclear, whether the effect on gene expression depends on the intronic short tandem repeat (STR) (CA)*_n_* splicing regulator or not. Here we investigated the STR (CA)*_n_* polymorphism in rs10886471 and further discussed its role in the T2DM risk of Chinese Hainan Island individuals. A total of 1164 subjects were recruited and classified into a normal fasting glucose (NFG) group, an impaired fasting glucose (IFG) group, an impaired glucose tolerance (IGT) group, and a T2DM group. STR (CA)*_n_* polymorphisms were detected through polymerase chain reaction and sequencing. Five intronic (CA)*_n_* alleles, (CA)*_15_* to (CA)*_19_*, were identified in *GRK5* rs10886471. Only the (CA)*_16_* allele was significantly associated with increased prediabetes and T2DM risk [odds ratio (OR)>1, *P*<0.05]. Conversely, multiple alleles without any (CA)*_16_* protected against prediabetes and T2DM (0<OR<1, *P*<0.05). In summary, rs10886471 acts as both an SNP and an STR. The rs10886471 intronic SNP causes *GRK5* overexpression the subsequent risk of T2DM may be due to the rs10886471 intronic STR (CA)*_n_* splicing enhancer. Further studies should focus on verifying these finding using a large sample size and analyzing the splicing mechanism of intronic (CA)*_n_* in rs10886471.

## Introduction

Type 2 diabetes mellitus (T2DM) is one of the most common diseases; it has a high incidence, numerous complications, high disability rate, low awareness rate, and heavy economic burden. Many countries pay heavy costs for T2DM every year[1]. Although the genetic heterogeneity of T2DM is associated with genetic and environmental factors, genetic polymorphism and susceptibility to T2DM remain largely unknown. About 20 genes and 60 genetic loci have been linked to T2DM susceptibility[2], [3], [4], [5], [6]. A recent study indicated that the T2DM susceptibility of Chinese Han populations, including East Asian populations, is significantly higher than those of Western populations. This increased T2DM susceptibility has been associated with G-protein–coupled receptor kinase 5 (*GRK5*) rs10886471, which is endemic to East Asian populations[3], [7]. The cis-expression quantitative loci (cis-eQTL) analysis and quantitative real-time RT-PCR showed that the rs10886471 SNP allele changes the transcription level of the *GRK5* gene[3], [5], but the mechanism remains unclear. Non-coding microsatellite polymorphism could act as a functional unit and interact with promoter SNPs during transcription regulation[8]. The rs10886471 is located in the intron region of *GRK5*. However, whether the effect on the gene expression of rs10886471 intronic SNP depends on the intronic (CA)*_n_* splicing regulator should be studied. We first report an intronic (CA)*_n_* repeat polymorphism in *GRK5* rs10886471 and susceptibility to T2DM.

## Methods

### Subjects

The inclusion criterion for subjects was age ranging from 35 years to 85 years old. The exclusion criteria were as follows: type 1 diabetes, recent acute disease, chronic inflammatory disease, infectious disease, and metabolic disease other than prediabetes and diabetes. Prediabetes and diabetes were diagnosed according to the diagnostic criteria[9]. The adult community residents (n = 1164, 584 men and 580 women) were recruited from Haikou City on Hainan Island from March 2011 to September 2011 using a multistage stratified cluster sampling design. The following clinical characteristics and information were recorded for each subject: age, gender, body mass index (BMI), systolic blood pressure (SBP), diastolic blood pressure (DBP), fasting plasma glucose (FPG), and 2-hour plasma glucose(2 h PG) in the oral glucose tolerance test (OGTT). The subjects were assigned into four groups based on blood glucose level: normal fasting glucose (NFG) group (n = 282), impaired fasting glucose (IFG) group (n = 287), impaired glucose tolerance (IGT) group (n = 293), and T2DM group (n = 302). The age composition did not differ by more than 5 years, and the gender composition ratio did not differ by more than 5%. Physical examination and blood biochemical testing were conducted for all subjects. GRK5 rs10886471 (CA)n polymorphism experiments were also performed from October 2011 to March 2013 as follow-up tests. Our study was considered and approved by Hainan medical ehtics committee on January 2011. Our study began after all participants provided written informed consent.

### Microsatellite polymorphisms detection

Genomic DNA was extracted from the peripheral blood using a BloodGen Mini kit (CWBiotech, Beijing, China). Microsatellite polymorphism was identified via PCR and sequencing. The primers were designed to amplify the 320 bp region of *GRK5* rs10886471. Information on the rs10886471 sequence is available online (http://www.ncbi.nlm.nih.gov/projects/SNP/snp_ref.cgi?rs=10886471#fasta). The forward primer was 5′-aagttcttccctgctagagaa-3′ and the reverse primer was 5′-ctctttttgttctaagtgaaaac-3′. PCR was performed under the following conditions: initial denaturation at 94°C for 5 min; followed by 33 cycles of denaturation at 94°C for 1 min, annealing at 53°C for 1 min, and extension at 72°C for 1 min; and a final extension at 72°C for 7 min. The reaction was performed at a final volume of 50 µl, which contained the basic reaction components. The PCR products were verified via 2.0% agarose gel electrophoresis and purified using a Quick Gel Extraction Kit (CWBiotech, Beijing, China). The purified PCR products were directly sequenced or ligated into a pGEM-T Easy Vector sequence (Shanghai Sangon Biotech Co. Ltd, China). The sequencing results were aligned with the intron region of the *GRK5* gene from GenBank (NM_005308.2) and were analyzed using the BioEdit software. Standard procedures and the latest scientific test specifications were strictly followed. Two people independently counted the alleles and discrepancies between the two examiners were resolved through repeat examinations of the samples.

### Statistical analysis

The microsatellite polymorphism was analyzed using the SSRHunter genetic profiler software. The (CA)*_n_* allelic frequencies were estimated through direct gene counting. Polymorphism information content (PIC) was calculated using the PIC-Calc0.6 software. A Pearson's chi-square test was used to count the variables and an ANOVA was used for mean comparisons. Forward stepwise regression was used for multivariate logistic regression analysis to estimate the strength of the associations of *GRK5* polymorphism with prediabetes and with T2DM. SPSS v17.0 was used for all statistical analysis. Differences with p values <0.05 were considered statistically significant, and all *p* values are two tailed.

## Results

### General data

**Table 1** summarizes the clinical characteristics and biochemical results of the subjects. The four groups did not significantly differ in terms of age and gender (*P*>0.05). However, waist circumference, BMI, SBP, DBP, FPG, and 2 h PG increased with the abnormal increase in blood glucose (IFG and IGT groups) and continued to increase with the blood sugar until it reached T2DM levels. The clinical parameters significantly differed between the four groups (all *P*<0.05).

**Table 1:** Clinical characteristics of the study subjects.

Parameters	NFG	IFG	IGT	T2DM	*P* ^a^
	(n = 282)	(n = 287)	(n = 293)	(n = 302)	
Gender					
Male	136 (48.23)	148 (51.57)	153 (52.22)	147 (48.68)	0.703
Female	146 (51.77)	139 (48.43)	140 (47.78)	155 (51.32)	
Age	63±3.51	67±2.72	69±3.86	68±1.99	0.992
Waist (m)	0.51±0.01	0.55±0.01	0.61±0.01	0.63±0.01	0.000
BMI (kg/m^2^)	20.79±1.30	23.37±0.44	27.18±0.47	26.35±0.42	0.000
SBP (mmHg)	110.12±5.82	119.59±2.90	125.47±2.1	128.51±3.79	0.001
DBP (mmHg)	72.39±4.32	90.11±2.79	99.91±2.7	102.89±2.15	0.000
FPG (mmol/L)	4.63±0.83	6.45±0.29	6.51±0.23	7.60±0.65	0.001
2 h PG (mmol/L)	5.09±1.37	6.49±0.65	8.76±1.23	12.08±0.59	0.000

Gender is expressed as n (%). All other parameters are expressed as mean ± S.D. BMI, body mass index; SBP, systolic blood pressure; DBP, diastolic blood pressure; FPG, fasting plasma glucose; 2 h PG, 2-hour plasma glucose in the OGTT. **^a^** Comparison of four groups via analysis of variance (continuous variables) or χ^2^-test (count variables).

### (CA)n polymorphism in rs10886471

**Figure 1:**
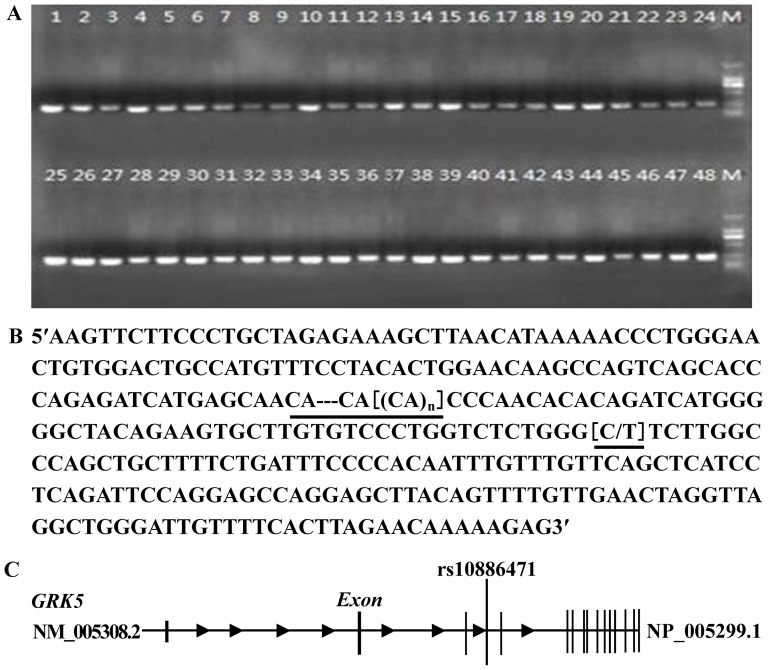
Intronic (CA)*n* polymorphism in *GRK5* rs10886471. (A) Amplified fragments in the *GRK5* rs10886471 studied region. The PCR products were electrophoresed in 2% agarose gel and were then photographed under UV light. Bands 1–12 were from the NFG group, bands 13–24 from were from the IFG group, bands 25–36 were from the IGT group, and bands 37–48 were from the T2DM group. The PCR marker was in lane M. (B) SNPSTR marker in *GRK5* rs10886471. PCR sequencing demonstrated a STR (CA)*_n_* with one tightly linked SNP (C/T) in *GRK5* rs10886471. (C) Sequence analysis using the physical map of *GRK5* rs10886471. The SNPSTR marker is located in the intron of *GRK5* rs10886471.

**Figure 2:**
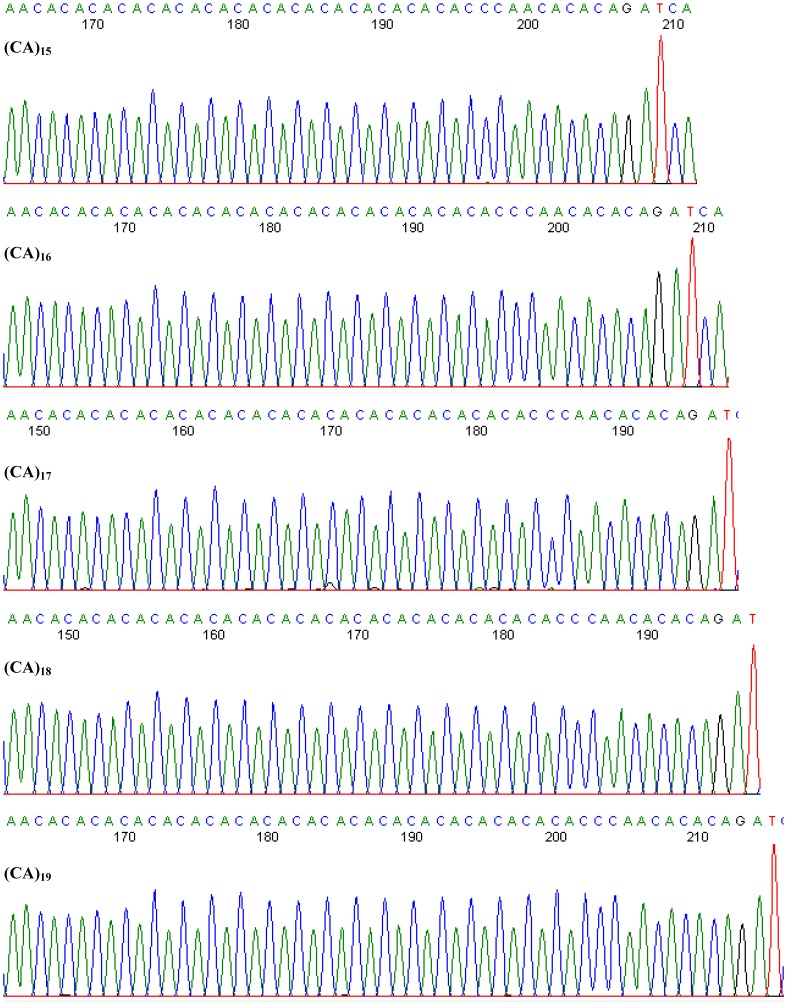
Detection of (CA)*_n_* alleles in *GRK5* rs10886471. The allelic length of the (CA)*_n_* repeats ranged from 15 to 19, as determined by automated fluorescence-based PCR sequencing.

CA repeat sequences are abundant in the human genome. Numerous studies have revealed that intronic (CA)*_n_* repeats could play a novel and generally important role in the splicing of enhancers or repressors during gene expression[10], [11], [12], [13]. Therefore, we extended the rs10886471 SNP analysis to study the short tandem repeat (STR) function. Genomic DNA from 1164 subjects was amplified via PCR and sequenced using primers specific for the rs10886471 studied region (about 320 bp) as shown in **Figure 1** and **2**).

### (CA)n allelic frequencies of rs10886471

**Figure 3:**
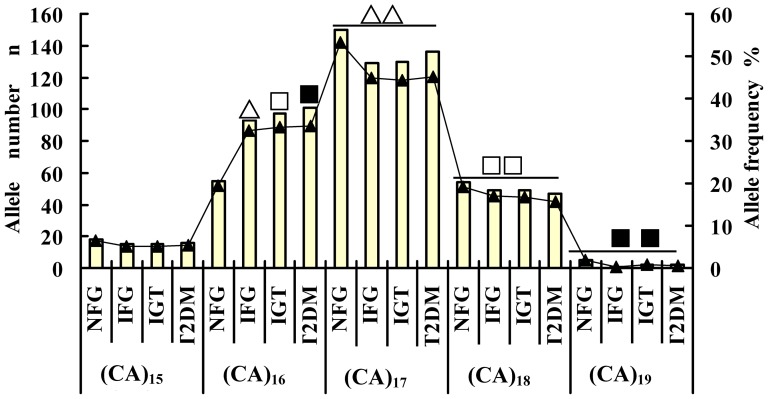
Frequency distribution of rs10886471 (CA)*_n_* alleles in the four groups. Not all comparisons are shown. The allelic frequencies of (CA)*_16_* in the IFG, IGT, and T2DM groups wasmuch higher than that in the NFG group (^△^ **X** **^2^** = 12.300, *P* = 0.000; ^□^ **X** **^2^** = 13.672, *P* = 0.000;^▪^ **X** **^2^** = 14.476,*P* = 0.000, respectively). The allelic frequency of (CA)*_16_* was significantly lower than that of (CA)*_17_*, but higher than those of (CA)*_18_* and (CA)*_19_* among the four groups (^△△^ **X** **^2^** = 16.190, *P* = 0.001; ^□□^ **X** **^2^** = 10.221,*P* = 0.017; ^▪▪^ **X** **^2^** = 8.265, *P* = 0.041, respectively).

The allelic frequencies are listed in **Figure 3** and **Table 2**, (CA)*_17_* had the highest allelic frequency in each group, followed (CA)*_16_*. However, the allelic frequencies of (CA)*_16_* and (CA)*_17_* were lower in the NFG group than in the IFG, IGT, and T2DM groups. The allelic frequencies of (CA)_15_, (CA)*_18_*, and (CA)*_19_* in the NFG group were higher than those in the IFG, IGT, and T2DM groups. The allelic frequency of (CA)*_16_* was significantly lower than that of (CA)*_17_*, but significantly higher than those of (CA)*_18_* and (CA)*_19_* among the four groups (χ^2^ = 16.190, *P* = 0.001; χ^2^ = 10.221, *P* = 0.017; and χ^2^ = 8.265, *P* = 0.041, respectively). The allelic frequency distributions of (CA)*_15_*, (CA)*_17_*, (CA)*_18_*, and (CA)*_19_* did not significant differ among the four groups [(CA)*_15_* (χ^2^ = 0.570, *P* = 0.903); (CA)*_17_* (χ^2^ = 6.096, *P* = 0.107); (CA)*_18_* (χ^2^ = 1.368, *P* = 0.713); (CA)*_19_* (χ^2^ = 3.889, *P* = 0.274), respectively]. By contrast, the frequency of (CA)*_16_* increased with the abnormally increasing blood glucose (IFG and IGT groups) and continued to increase with blood sugar until it reached T2DM levels. The allelic frequencies of (CA)*_16_* in the IFG, IGT, and T2DM groups were much higher than in the NFG group (χ^2^ = 12.300, *P* = 0.000; χ^2^ = 13.672, *P* = 0.000; χ^2^ = 14.476, *P* = 0.000, respectively).

**Table 2:** Association of GRK5 intronic (CA)_n_ repeat polymorphisms with prediabetes and T2DM (n = 1164).

Allele	NFG	IFG				IGT				T2DM			
	n	n	?^2^	OR (95% CI)	*P*	n	?^2^	OR (95% CI)	*P*	n	?^2^	OR (95% CI)	*P*
(CA)*_17_* a	150	129		1.000		130		1.000		136		1.000	
(CA)*_15_*	18	15	0.015	0.955(0.463–1.971)	0.901	15	0.015	0.955(0.463–1.971)	0.901	16	0.005	0.974(0.478–1.986)	0.942
(CA)*_16_*	55	93	10.102	1.938(1.289–2.915)	0.001	97	11.547	2.021(1.347–3.034)	0.001	101	11.595	2.012(1.345–3.009)	0.001
(CA)*_18_*	54	49	0.029	1.04(0.661–1.635)	0.865	49	0.029	1.04(0.661–1.635)	0.865	47	0.042	0.954(0.605–1.503)	0.838
(CA)*_19_*	5	1	1.787	0.229(0.026–1.987)	0.181	2	0.851	0.458(0.087–2.403)	0.356	2	0.953	0.438(0.084–2.296)	0.329
(CA)*_16_* a	55	93		1.000		97		1.000		101		1.000	
Lacking (CA)*_16_* allele ^b^	227	194	11.761	0.51(0.347–0.75)	0.001	196	13.252	0.492(0.336–0.721)	0.001	201	14.024	0.484(0.331–0.708)	0.000

a Reference allele; **^b^**Lacking the (CA)*_16_* allele and contains (CA)*_15_*, (CA)*_17_*,(CA)*_18_*, and (CA)*_19_*; OR, odds ratio; 95%CI, 95% confidence interval.

### The PIC values of the rs10886471 (CA)n alleles

The PIC values of the rs10886471 (CA)*_n_* alleles in the NFG, IFG, IGT, and T2DM groups were 0.6146, 0.6233, 0.6291, and 0.6327, respectively. The PICs of the four groups all exceeded 0.5, which indicates that the *GRK5* (CA)*_n_* repeats exhibited genetic polymorphism. The PIC of each group did not significantly deviate from the Hardy–Weinberg equilibrium.

### Association of GRK5 (CA)n polymorphisms with prediabetes and T2DM

Logistic regression analysis was conducted on the alleles and the results are presented in **Table 2**. The NFG group was designated as the control group, whereas the three remaining groups were designated as the case groups and classified as dependent variables (NFG = 0, IFG = 1, IGT = 2, and T2DM = 3). Each allele was classified as an independent variable. The statistical significance of the inclusion criteria was set to *P*>0.05, whereas that for the exclusion criteria was set to *P*<0.10. **Table 2** shows the association of intronic (CA)*_n_* repeat polymorphisms with prediabetes and T2DM risk. Using the most common (CA)*_17_* allele as a reference for estimating the strength of the association, allele (CA)*_16_* was significantly associated with increased risk of prediabetes and T2DM [IFG, OR (95% CI) = 1.938 (1.289–2.915), *P* = 0.001; IGT, OR (95% CI) = 2.021(1.347–3.034), *P* = 0.001; T2DM, OR (95% CI)  = 2.012 (1.345–3.009), *P* = 0.001, respectively]. The other alleles were not significantly associated with abnormal blood glucose (*P*>0.05). The NFG group was designated as the control group, whereas the other three groups were designated as the case groups. All alleles without (CA)*_16_* that included (CA)*_15_*, (CA)*_17_*, (CA)*_18_*, and (CA)*_19_* were classified as one multiple allele. The (CA)*_16_* allele was used as a reference. The multiple alleles were negatively correlated significantly with the IFG, IGT, and T2DM groups [IFG, OR (95% CI)  = 0.510 (0.347–0.750), *P* = 0.001; IGT, OR (95% CI)  = 0.492 (0.336–0.721), *P* = 0.001; T2DM, OR (95% CI)  = 0.484 (0.331–0.708), *P* = 0.000, respectively].

## Discussion

**Table 1** shows that the biochemical indices increased with the abnormal increase in blood glucose from IFG and IGT levels to T2DM levels. The interaction between the indices and the genetic polymorphism requires further research. SNPs and STRs are presently the two main genetic markers. The SNPSTR, which is a STR with one or more tightly linked SNPs, is a relatively new type of marker[14]. A previous study reported that the rs10886471 SNP is a risk marker T2DM[3]. The mRNA levels of the *GRK5* gene in the peripheral blood of the T2DM group was significantly higher than that in the controlled group, which suggests that the allelic frequency of the rs10886471 SNP affects the *GRK5* gene transcription level[3], [15]. However, how the rs10886471 intronic SNP affects transcription remains uncertain. Intronic SNPs may play a role by directly affecting gene expression or through linkage disequilibrium (LD) with another SNP[16].

Our study shows that rs10886471 is an SNP with a tightly linked STR marker. The increasing frequency of rs10886471 STR (CA)*_16_* is consistent with the increase in blood glucose. The frequency of the (CA)*_16_* allele was significantly lower than that of the (CA)*_17_* allele, but higher than that of alleles (CA)*_18_* and (CA)*_19_* among the four groups (all *P*<0.05). The logistic regression models also showed that the (CA)*_16_* allele is the only risk factor significantly associated with abnormal blood glucose (OR>1, *P*<0.05). The multiple alleles without any (CA)*_16_* were significantly correlated negatively with prediabetes and T2DM (0<OR<1, *P*<0.05). Therefore, the (CA)*_16_* allele of rs10886471 may contribute to the risk of developing T2DM, but multiple alleles without any (CA)*_16_* may be protective. Consequently, the (CA)*_n_* polymorphism of *GRk5* rs10886471 has a risk-protective yin–yang effect against prediabetes and T2DM. Our STR study combined with previously reports on rs10886471 SNP shows that the mechanism by which rs10886471 intronic SNP influences gene expression may differ from direct effect or LD with another SNP.

**Figure 4:**
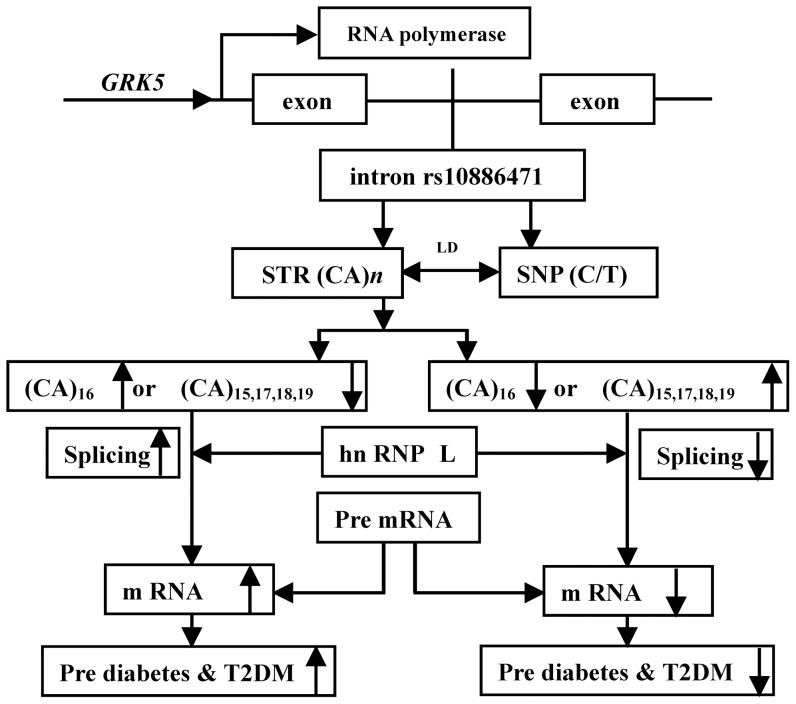
The mechanism of intronic (CA)*_n_* splicing regulator in rs10886471. The heterogeneous nuclear ribonucleoprotein L (hnRNP L) is specifically bound to diverse CA elements. ^10, 13^ It contains four RNA recognition motifs (RRMs) that bind to CA repeats. The crystal structures of hnRNP L RRMs at 2.0 and 1.8 Å has been elucidated. ^20^ The intronic (CA)*_n_* repeats in *GRK5* rs10886471 act as splicing enhancers or repressors and their yin–yang effect on T2DM depends on the CA repeat number. Intronic SNPs that affect gene expression may be mediated by LD with intronic STR (CA)*_n_* regulators.

The most common cause of STR (CA)*_n_* repeats is replication slippage, which is caused by mismatches between DNA strands[17]. Numerous associations of variants with phenotypes cannot be elucidated using exonic variants; this limitation highlights the need for intronic variants[18]. Changes in length of STR (CA)*_n_* repeats within cis-regulatory regions can also change gene expression. As previously reported, microsatellites are predictors of nucleotide diversity and divergence[19], and (TG/CA)*_n_* repeats are present in the regulation of transcription from disease-related genes such as epidermal growth factor receptor, hydroxysteroid (11-beta) dehydrogenase 2, interferon-gamma, and *CD154* [10], [11], [12], [13]. These mounting findings suggest that rs10886471 intronic SNP that causes *GRK5* overexpression and the subsequent risk of T2DM may be due to the involvement of intronic STR (CA)*_n_* in splicing (**Figure 4**).

*GRK5* affects insulin signal transduction pathways. The (CA)*_16_* of rs10886471 changes the *GRK5* gene transcription level via splicing code. *GRK5* phosphorylates G protein–coupled receptors (GPCRs), which are signal transduction receptors involved in glucose metabolism[20], [21], [22]. After phosphorylation by *GRK5*, GPCRs negatively regulate the effects of the glucose metabolic signal, and causes abnormal blood glucose and diabetes[22], [23]. Previous studies have reported that *GRK5* and GPCR (class A) are related to cardiovascular and cerebrovascular diseases[24], [25]. Current studies have shown that GPCRs (class B) are promising therapeutic targets that may aid in the design of new small-molecule drugs for metabolism diseases[26], [27]. However, the signal transduction pathway of GRK5–GPCR (class B) in T2DM remains unknown. The intronic (CA)*_n_* splicing regulator in *GRK5* expression provides new insight into the transduction mechanism of T2DM.

In summary, GRK5 rs10886471 acts as both an SNP site and an STR site, i.e., an SNPSTR marker. The rs10886471 STR has five (CA)*_n_* alleles and exerts a yin–yang effect on T2DM.The yin–yang effect may be dependent on the number of STR (CA)*_n_* repeats. The rs10886471 intronic SNP that causes *GRK5* overexpression and the subsequent risk of T2DM may caused by the rs10886471 intronic STR (CA)*_n_* splicing enhancer. Further studies should focus on a comprehensive association analysis between the *GRK5* rs10886471 SNP and STR with a large sample size. In addition, the mechanism by which the intronic (CA)*_n_* splicing code regulates the signal transduction of GRK5–GPCR (class B) should be elucidated.
